# Examination of ethical intelligence and cognitive flexibility of nurses and their role in predicting the level of patient privacy protection

**DOI:** 10.1186/s12912-024-02153-y

**Published:** 2024-07-23

**Authors:** Gholamhossein Mehralian, Ali Reza Yusefi, Jamshid Bahmaei, Shima Bordbar

**Affiliations:** 1 University of Exeter Business School, Exeter, UK; 2 School of Medical Sciences, Sirjan School of Medical Sciences, Sirjan, Iran; 3 Department of Public Health, Behbahan Faculty of Medical Sciences, Behbahan, Iran; 4 Health Human Resources Research Center, School of Health Managemet and Information Sciences, Shiraz University of Medical Sciences, Shiraz, Iran

**Keywords:** Ethical intelligence, Cognitive flexibility, Privacy, Nurse, Patient, Hospital

## Abstract

**Introduction:**

Possessing ethical intelligence and cognitive flexibility can play a significant role in the acceptable performance of nurses. Furthermore, respecting the privacy of patients should always be a primary ethical principle that nurses focus on. This study aimed to investigate the ethical intelligence and cognitive flexibility of nurses and their role in predicting the level of patients’ privacy observance. Also, determining the overall status of patient privacy protection and its two domains, namely human dignity domain and maintaining personal privacy, were specific objectives of this study.

**Methods:**

This cross-sectional descriptive-analytical study was conducted in 2022. A sample of 340 nurses and 1067 patients from teaching hospitals affiliated with Shiraz University of Medical Sciences in southern Iran were selected. Standard questionnaires of ethical intelligence and cognitive flexibility were used for nurses, and a privacy observance questionnaire was used for patients. Data were analyzed using t-tests, ANOVA, Pearson correlation coefficient, and multiple linear regression with the SPSS23 software.

**Results:**

The mean score of ethical intelligence and cognitive flexibility for nurses was 98.33 ± 18.06 (out of 200) and 74.56 ± 16.76 (out of 140), respectively. The mean score of patients’ privacy observance was 79.74 ± 14.53 (out of 150). The results of multiple linear regression showed that the dimensions of perseverance and assertiveness towards rights (*β* = 0.540, *p* < 0.001), action based on principles, values, and beliefs (*β* = 0.454, *p* < 0.001), responsibility towards personal decisions (β = 0.410, *p* < 0.001), accepting responsibility for serving others (*β* = 0.393, *p* < 0.001), ability to forgive one’s mistakes (*β* = 0.301, *p* = 0.001), ability to forgive others’ mistakes (*β* = 0.287, *p* = 0.002), honesty (*β* = 0.275, *p* = 0.004), acknowledgment of mistakes and failures (*β* = 0.263, *p* = 0.005), commitment to promises (*β* = 0.242, *p* = 0.005), and interest in others (*β* = 0.237, *p* = 0.01) from the dimensions of ethical intelligence, as well as the dimensions of perceived control (*β* = 0.580, *p* < 0.001), perception of multiple solutions (*β* = 0.511, *p* < 0.001), and perception of justifications (*β* = 0.373, *p* < 0.001) from the dimensions of cognitive flexibility had a positive and significant effect on the level of patients’ privacy observance.

**Conclusion:**

Ethical intelligence and cognitive flexibility of nurses and the level of patient privacy protection were estimated to be at a moderate level. Also, the level of ethical intelligence and cognitive flexibility of nurses played a predictive role in the level of patients’ privacy observance. It is suggested that hospital managers and policymakers enhance nurses’ ethical intelligence and cognitive flexibility through educational, welfare, managerial, motivational, and job-related programs, thereby improving the status of patient privacy protection.

## Introduction

One of the most important aspects of health services is the ethics and adherence to ethical principles by the providers of these services [1]. Since the promotion of health and prevention of future diseases in any society is the responsibility of medical professionals, it is necessary to adopt the best educational paths to maintain health security [2]. Education is not only about theoretical and practical lessons but also requires teaching a series of principles that are the foundation of every matter; one of these principles is ethics and ethical considerations [3]. In treatment, to achieve the main goal of the care team, which is the health of the patient, the patient’s trust in the treatment team is essential, and the prerequisite for trust is adherence to ethical principles. Therefore, to enhance community health, it is necessary for ethical principles to be seriously observed among healthcare staff to achieve therapeutic goals by gaining the trust of patients [4].

A hospital is an organizational structure with specialized personnel having diverse orientations participating in various aspects of patient care [5]. One of the most significant service providers in this organization is nurses [6]. Since nurses constantly face numerous ethical decisions throughout the care and treatment of patients, they are more susceptible to ethical issues in their workplace compared to other healthcare professionals [7]. Ethical intelligence, as a foundation for proper human performance, is one dimension of intelligence and can predict behavior [8]. It entails the ability to perceive right from wrong, possess strong moral beliefs, and act on them [9]. Ethical intelligence plays a constructive role in improving the job and psychological status of nurses [10]. Nurses with high ethical intelligence can discern pain and suffering in others, control ruthlessness and temptation within themselves, listen impartially, accept differences, explore various human values, reject unethical options, fight injustice, understand others, and treat them respectfully [11]. Nurses with high ethical intelligence can ensure the health institutions’ well-being and optimize their position [12]. Furthermore, by enhancing their ethical intelligence, nurses can effectively interact with colleagues and patients [13]. High ethical intelligence of nurses helps them adhere to ethical standards in their performance and positively impacts nursing services, the quality of nurses’ work, and the improvement of patients’ recovery processes [14]. Having desirable ethical intelligence and adhering to ethical principles enable patients to receive nursing care with greater confidence and trust [8]. Any breach of ethical standards by nurses can affect even the most scientific and best nursing care [12]. Additionally, ethics and ethical values derived from appropriate ethical intelligence in nurses can reduce costs associated with control and improvement of relationships, increase understanding, and reduce conflicts [15]. On the other hand, the results of some studies indicate the impact of ethical intelligence on certain variables. A study by Sadeghi and colleagues demonstrated a positive and significant relationship between nurses’ ethical intelligence and patient satisfaction, indicating that patients were more satisfied with nurses who had higher ethical intelligence [16]. The findings of a review study by Yousefi and colleagues indicated that identifying ethical intelligence is considered a fundamental and essential requirement in nursing, as becoming a good nurse is not solely dependent on theoretical knowledge and clinical skills but also on the development of ethical experiences in the application of this knowledge and ethical responsibility [17]. Therefore, adherence to ethical criteria in the performance of nurses is more sensitive and important in comparison to other aspects of care, as ethical behavior combined with nurses’ responsibility can be considered an effective factor in the improvement and recovery of the health of patients and beneficiaries [18].

In today’s rapidly changing world, another determinant factor for desirable nursing performance in the hospital environment is having an adequate level of cognitive flexibility [19]. Cognitive flexibility is the ability to adapt cognitive and behavioral strategies in response to constant environmental changes [20]. In fact, cognitive flexibility refers to the ability to understand the controllable aspects of challenging and complex situations, provide interpretations and diverse solutions for these situations, and not avoid challenging situations [21]. This concept is defined as a dynamic process, responsible for creating positive adaptation despite opposing experiences in an individual [22]. New theories view cognitive flexibility as a multidimensional structure that includes fundamental variables such as personality, specific skills, and problem-solving skills [23]. Cognitive flexibility also plays an important role in health-related behaviors [24]. Flexible individuals are curious both internally and externally, enriching their lives with a variety of experiences [25]. They enjoy and seek out new opportunities, actively confronting and exploring different types of experiences [25]. Nurses belong to the category of professionals responsible for the health and well-being of individuals, bearing a set of relatively challenging responsibilities in the fields of physical health and mental hygiene, facing a number of physical and psychological stressors in the workplace, including long working hours, heavy workload, required and unrequired overtime, among others. Long-term activity of nurses in such an environment can lead to their disorder and incorrect performance [26]. The result of this condition may endanger their physical and mental health, including the risk of developing cardiovascular and gastrointestinal diseases, reduced immunity, chronic fatigue, job burnout, and depression [27]. Indirect consequences include a reduction in the quality of care provided by nurses, more frequent medical errors, and thus decreased patient satisfaction [28]. In this context, cognitive flexibility can play a significant role in managing and controlling these conditions [29]. According to some studies, nurses’ cognitive flexibility can help improve and enhance their quality of work and personal life, thereby ensuring the provision of high-quality services by nurses in work environments such as hospitals [30–32]. Studies conducted in Iran have shown that the average cognitive flexibility score (with a score range of 20–140) of nurses working in hospitals ranges from 73.66 [33] to 93.32 [34], indicating the need for improvement in this essential component among nurses.

In hospital environments, one of the most critical relationships that is consistently emphasized and stressed is the nurse-patient relationship, as this relationship plays a significant role in the recovery of patients’ health and the provision of quality services [35]. According to previous studies, among the factors that can facilitate and enhance this relationship, the ethical intelligence of nurses [36, 37], and their cognitive flexibility [29] can be mentioned. Nurses with desirable ethical intelligence and cognitive flexibility are likely to establish a comprehensive relationship with their patients, taking into account the preservation of human dignity and understanding different conditions and situations from simple to complex, guiding them on the path to recovery [29, 36, 37]. Alongside other factors, what matters on this path is the preservation of patients’ privacy. The need to preserve privacy is one of the fundamental human rights [38], and the human personality, in terms of beliefs, culture, moral standards, plays a significant and fundamental role in the recovery from illness [39]. Therefore, privacy must be considered as one of the key concepts in nursing [38]. Patients need to feel comfortable revealing information to nurses and other healthcare professionals, and without trust in them, they may refrain from giving essential information for their care or distort information [40]. In other words, the need to preserve privacy and, consequently, the preservation of dignity is one of the fundamental needs of patients. Since one of the critical aspects of nursing is striving to meet the needs of beneficiaries, nurses, along with other healthcare team members, should pay full attention to the positive and respectful behavior of beneficiaries to meet their four essential needs: solitude, independence, security, and identity. This is because preserving privacy can have consequences such as fostering positive relationships with employees, creating a sense of security, reducing the duration of hospitalization by reducing psychological problems caused by privacy violations [41]. Based on studies, nearly one-fourth of admitted patients in hospitals report that their privacy has not been respected [42]. Findings from a study by Sadeghi indicate that privacy was not respected in one-third of cases in Iranian hospitals [43]. A study by Kuzu and colleagues in Turkey showed that the right to solitude and personal privacy of patients was preserved in 68.1% of cases [44]. The results of Erdil and Korkmaz also revealed that some patients’ boundaries were disregarded [45]. The findings of a study by Nayeri and colleagues in Iran indicated that respecting privacy was weak to moderate in 50.6% of patients [46].

A review of past studies shows that the three variables of ethical intelligence, cognitive flexibility of nurses, and patient privacy protection have mostly been examined separately, and few studies have simultaneously examined two or all three mentioned variables. Also, Considering the importance of ethical intelligence and cognitive flexibility of nurses in the hospital environment to provide quality health services, accelerate the treatment and recovery process of patients, as well as the significant role that respecting patients’ privacy has in their satisfaction and comfort during their hospitalization, this study aimed to examine the ethical intelligence and cognitive flexibility of nurses and their role in predicting the extent of respecting patients’ privacy.

## Methods

### Design and setting

This cross-sectional descriptive-analytical study was conducted in teaching hospitals affiliated with Shiraz University of Medical Sciences in southern Iran from December to May 2022.

### Participants

The population of this research included hospitalized patients and nurses working in teaching hospitals affiliated with Shiraz University of Medical Sciences, including 10 hospitals.

The required sample size for patients was estimated at 1067 individuals, considering a 95% confidence level and a 50% prevalence rate (*P* = 0.05), using the following formula.

$$n = {{Z_{1 - {\alpha \over 2}}^2P{\rm{(1}} - P{\rm{)}}} \over {{d^2}}}$$

The inclusion criteria for patients included willingness to cooperate with the researcher, being above 18 years of age, a minimum stay of 48 h in the hospital, and not having a diagnosed mental disorder by the attending physician. The exclusion criteria included inability to respond, insufficient alertness, and unwillingness to participate in the study. Thus, patients with mental problems recorded in their files and diagnosed by a doctor were excluded from the study. Also, patients who, due to physical conditions, lacked sufficient consciousness (including lack of eye response, verbal response, and motor response) were excluded from the study.

After determining the sample size, the number of hospitalized patients in each hospital was first determined, and using stratified sampling, the sample size of patients in each hospital was specified. Then, each hospital was visited, and to select the desired sample, the number of hospitalized patients in each ward was first determined, and based on the stratified sampling method, the sample size was distributed among the hospital wards. Finally, after determining the number of patients in each ward in each hospital, patients were randomly selected and included in the study.

Additionally, based on the population of nurses, which was 2943 individuals, the required sample size for examination was estimated at 340 individuals with a 5% margin of error, using the following formula. Then, by dividing 340 by 2943 and multiplying the obtained number by the number of nurses in each hospital, the required sample size in each hospital was determined. In Table 1, the names of the hospitals under investigation, the total number of nursing staff, and the sample size in each hospital are separately mentioned.

$$n = {{{{{z^2}pq} \over {{d^2}}}} \over {1 + {1 \over N}\left( {{{{z^2}pq} \over {{d^2}}} - 1} \right)}}$$

P = q = 0.5

d = 0.05

z = 1.96

N = 2943

**Table 1:** The number of nurses studied by each hospital

Hospitals	Total number of nurses	Sample size
Namazi	1047	121
Faghihi	491	56
Hazrat Ali Asghar	197	23
Chamran	264	30
Hazrat Zeinab	145	17
Khalilei	74	9
Hafez	117	14
Ib Sina	71	8
Rajaei	441	51
Dastgheib	96	11
Total	**2943**	**340**

Inclusion criteria for nurses included willingness to participate in the study, work experience of at least one year in a hospital and employment in various clinical departments of the hospital.

The exclusion criteria consisted of unwillingness to participate in the study, history of high stress and discomfort (for example, the death of a loved one, divorce, etc.) during the past month since participating in the study and working in non-clinical departments such as administrative and financial departments of the hospital.

In each hospital, the selection of nurses began by determining the number of nurses in each department. Subsequently, proportional stratified sampling was employed, and nurses were randomly chosen based on their personnel code using a table of random numbers. These selected nurses then participated in the study.

### Instruments

The data collection instruments included the Ethical Intelligence Questionnaire [47, 48], Cognitive Flexibility Questionnaire [49, 50], and Patient Privacy Compliance Questionnaire [51].

The first part of the Ethical Intelligence and Cognitive Flexibility questionnaires included demographic information about the nurses, such as age, gender, education level, work experience, employment status, marital status, the number of shifts per month, and the number of patients under their care. The first part of the Patient Privacy Compliance Questionnaire included demographic information about the patients, such as age, gender, place of residence, marital status, education level, and income level.

The Ethical Intelligence questionnaire by Lenik and Keel (2005) consists of 40 questions assessing the nurses’ ethical intelligence in ten dimensions: operating based on principles, values, and beliefs (4 items), honesty (4 items), resilience and advocacy for rights (4 items), commitment to promises (4 items), personal decision-making responsibility (4 items), admitting mistakes and failures (4 items), accepting responsibility for serving others (4 items), being actively interested in others (4 items), the ability to forgive one’s own mistakes (4 items), and the ability to forgive mistakes of others (4 items). For each item, a Likert scale with responses ranging from “never” (score 1) to “always” (score 5) was used. This questionnaire has been used in several studies in Iran, and its reliability has been reported with a Cronbach’s alpha coefficient of 0.89 after translation, content and face validity confirmation, and retest reliability assessment [47]. According to the score range (40–200), the state of ethical intelligence was classified and ranked as poor (40–79), average (80–119), good (120–159) and very good (160–200) [48].

The Cognitive Flexibility Questionnaire by Dennis & Vander Wal (2010) is a self-report tool consisting of 20 short questions with three dimensions: control (8 items), perception of multiple solutions (10 items), and perception of justifications (2 items) [49]. The scoring is based on a 7-point Likert scale (strongly disagree, disagree, somewhat disagree, neutral, somewhat agree, agree, strongly agree) from 1 to 7. This questionnaire assesses an individual’s cognitive flexibility in clinical and non-clinical settings and has been validated in Iran with a test-retest reliability coefficient of 0.71 and a Cronbach’s alpha coefficient of 0.90 [50]. The scoring range of this questionnaire is between 20 and 140, based on which the cognitive flexibility status of nurses is classified as poor (20–49), average (50–79), good (80–109), and very good (110–140) [50].

The Patient Privacy Compliance Questionnaire consists of 30 questions, with 12 questions related to how the nurses respect the human dignity domain and 18 questions related to how they respect the personal space of patients [51]. The scoring for questions with a positive aspect was as follows: always (score 5), often (score 4), sometimes (score 3), rarely (score 2), and never (score 1). For questions with a negative aspect (lack of respect), the scoring was reversed. The validity and reliability of this questionnaire have been confirmed in Iran, with a reported Cronbach’s alpha coefficient of 0.88 [39]. According to the scoring range of this questionnaire (30–150), the status of patients’ privacy compliance is ranked poor (30–59), average (60–89), good (90–119) and very good (120–150) [51].

### Procedures and statistical analysis

To collect data, two researchers (ARY & SB) visited the selected hospitals on different days of the week during morning, evening, and night shifts. Participation in the study and completion of the questionnaires were entirely voluntary and only done if the individuals expressed willingness. After explaining the research objectives and ensuring the confidentiality of responses, verbal consent was obtained from the participants. The questionnaires were then distributed and collected in person on the same day. Some patients requested assistance from the research team (ARY & SB) to complete the surveys.

The collected data were entered into the SPSS software version 23. Pearson correlation coefficients were used to examine the correlation between ethical intelligence, cognitive flexibility of nurses, patient privacy compliance, and the age of nurses and patients and the work experience of nurses. Independent t-tests were used to assess the mean differences in the three main research variables based on the gender of nurses and patients, marital status, and education level of nurses, as well as the place of residence of patients. Analysis of variance (ANOVA) was used to investigate the differences in the mean scores of ethical intelligence, cognitive flexibility of nurses, and patient privacy compliance based on employment status, the number of shifts per month, and the number of patients under the care of nurses, as well as the marital status, education level, and income level of patients. Finally, multiple linear regression was used to examine the simultaneous impact of different dimensions of nurses’ ethical intelligence and cognitive flexibility on the level of patient privacy compliance.

## Results

According to the descriptive findings, the majority of patients included in the study were in the age group of 20–35 years (35.80%), male (56.98%), urban residents (55.58%), married (47.71%), with a diploma or higher education (64.94%), and with a monthly income level of ten to twenty million Iranian Rials (51.45%). There was a statistically significant positive correlation between the age of patients and their level of patient privacy compliance (*p* = 0.04). As patients’ age increased, their level of patient privacy compliance also increased. The mean score of patient privacy compliance was significantly higher among women (45.82 ± 15.31) compared to men (42.02 ± 13.65), urban residents (46.29 ± 16.14) compared to rural residents (41.55 ± 14.28), and those with a diploma or higher education (44.62 ± 14.16) compared to others. Furthermore, with an increase in the monthly income level of patients, the mean score of patient privacy compliance significantly increased (*p* < 0.05) (Table 2).

**Table 2:** Frequency distribution of patients (n = 1067)

Variables	Category	Frequency (Percent)	The patient privacy compliance
Age (year)	< 20 20–35 36–50 > 50	129 (12.09) 382 (35.80) 342 (32.05) 214 (20.06)	***p***_**=**_ **0.04** ^**a**^ *r*_=_0.526^b^
Gender	Male Female	608 (56.98) 459 (43.02)	***p***_**=**_ **0.001** ^**a**^ *t*_=_3.286^c^
Place of residence	Rural Urban	474 (44.42) 593 (55.58)	***p***_**=**_ **0.02** ^**a**^ *t*_=_2.785^c^
Marital status	Single Married Divorced Widowed	474 (44.42) 509 (47.71) 46 (4.31) 38 (3.56)	*p*_=_0.22^a^ *F*_=_1.013^d^
Level of education	Illiterate Primary school Middle school Diploma and higher	61 (5.72) 134 (12.56) 179 (16.78) 693 (64.94)	***p***_**=**_ **0.01** ^**a**^ *F*_=_2.863^d^
Income Level (per month)	No income 10–20 million Rials 21–30 million Rials > 30 million Rials	381 (35.71) 549 (51.45) 102 (9.56) 35 (3.28)	***p***_**=**_ **0.002** ^**a**^ *F*_=_4.127^d^
**Total**	-----	**1067 (100)**	-----

^a^*P-Value*; Correlation is significant at the 0.05 level

^b^*r*; Pearson Correlation Coefficient

^c^*t*; T-Test

^d^*F*; Test ANOVA

Furthermore, the average age of participating nurses was 30.23 ± 6.46 years, with the majority (52.65%) being under 30 years old. The average work experience was 7.23 ± 6.45 years, with most (63.82%) having less than 10 years of experience. The majority of participants were female (60.88%), held a bachelor’s degree (87.06%), worked more than 20 shifts per month (70%), and had more than 3 patients under their care during each shift (80.59%).

Statistically significant correlations were observed between the age of nurses and their ethical intelligence. As nurses’ age increased, their ethical intelligence also increased (*p* = 0.04). The average score of ethical intelligence among female nurses (100.54 ± 19.25), married nurses (99.73 ± 18.73), and nurses with a master’s degree (99.87 ± 17.41) was higher than others, and this average score decreased with an increase in the number of shifts per month and the number of patients under their care during each shift.

Significant correlations were also found between the age and work experience of nurses and their cognitive flexibility. As age and work experience increased, the cognitive flexibility of nurses also increased (*p* < 0.05). Moreover, the average score of cognitive flexibility among nurses varied significantly based on their educational level (*p* = 0.04), the number of shifts per month (*p* = 0.01), and the number of patients under their care during each shift (*p* = 0.003). Nurses with a master’s degree had a higher average score of cognitive flexibility (77.38 ± 17.24) compared to those with a bachelor’s degree (71.74 ± 15.46), and this average score decreased with an increase in the number of shifts per month and the number of patients under their care during each shift (Table 3).

**Table 3:** Frequency distribution of nurses (n = 340)

Variable	Categories	Frequency (Percent)		
			Ethical Intelligence	Cognitive Flexibility
Age (years)	30˃ 30–40 40˂	179 (52.65) 117 (34.41) 44 (12.94)	***p***_**=**_ **0.04** ^**a**^ *r*_=_0.472^b^	***p***_**=**_ **0.01** ^**a**^ *r*_=_0.663^b^
Work experience (years)	10˃ 10–20 20˂	217 (63.82) 101 (29.71) 22 (6.47)	*p*_=_0.08^a^ *r*_=_0.381^b^	***p***_**=**_ **0.02** ^**a**^ *r*_=_0.549^b^
Gender	Male Female	133 (39.12) 207 (60.88)	***p***_**=**_ **0.005** ^**a**^ *t*_=_4.109^c^	*p*_=_0.17^a^ *t*_=_1.118^c^
Marital status	Single Married	146 (42.94) 194 (57.06)	***p***_**=**_ **0.02** ^**a**^ *t*_=_2.843^c^	*p*_=_0.14^a^ *t*_=_0.738^c^
Level of education	Bachelors’ Master’s	296 (87.06) 44 (12.94)	***p***_**=**_ **0.001** ^**a**^ *t*_=_4.365^c^	***p***_**=**_ **0.04** ^**a**^ *t*_=_2.694^c^
Type of employment	Formal Contractual Contract-Based Project Corporate	62 (18.23) 71 (20.88) 54 (15.88) 127 (37.36) 26 (7.65)	*p*_=_0.21^a^ *F*_=_1.271^d^	*p*_=_0.32^a^ *F*_=_0.419^d^
Number of shifts per month	10˃ 10–20 20˂	55 (16.18) 47 (13.82) 238 (70)	***p***_**=**_ **0.03** ^**a**^ *F*_=_2.554^d^	***p***_**=**_ **0.01** ^**a**^ *F*_=_3.396^d^
Number of patients under observation in each work shift	2 3 3˂	43 (12.65) 23 (6.76) 274 (80.59)	***p***_**=**_ **0.001** ^**a**^ *F*_=_5.227^d^	***p***_**=**_ **0.003** ^**a**^ *F*_=_4.862^d^
**Total**	**------**	**340 (100)**	**------**	**------**

^a^*P-Value*; Correlation is significant at the 0.05 level

^b^*r*; Pearson Correlation Coefficient

^c^*t*; T-Test

^d^*F*; Test ANOVA

According to Table 4, the overall average scores for nurses’ ethical intelligence and cognitive flexibility were 98.33 ± 18.06 (out of 200) and 74.56 ± 16.76 (out of 140), respectively. These scores indicate a moderate level of these two variables among the nurses in the study.

**Table 4:** Mean and standard deviation of moral intelligence and cognitive flexibility and their dimensions from the point of view of the studied nurses

Variables	Dimensions	Score range	Mean	Standard deviation
**Ethical intelligence**	Operating based on principles, values, and beliefs	4–20	10.08	2.04
	Honesty		10.16	1.86
	Resilience and advocacy for rights		10.07	2.10
	Commitment to promises		9.85	1.65
	Personal decision-making responsibility		10.21	2.14
	Admitting mistakes and failures		9.42	1.95
	Accepting responsibility for serving others		9.97	1.53
	Being actively interested in others		9.50	1.77
	The ability to forgive one’s own mistakes		9.49	2.01
	The ability to forgive mistakes of others		9.58	2.28
	**Total**	**40–200**	**98.33**	**18.06**
**Cognitive flexibility**	Control	2–14	10.95	3.67
	Perception of multiple solutions	10–70	34.89	9.42
	Perception of justifications	8–56	28.72	6.54
	**Total**	**20–140**	**74.56**	**16.76**

The overall average scores for patients’ privacy preservation regarding both the preservation of human territory and the preservation of personal space were 38.26 ± 4.25 (out of 60) and 41.48 ± 6.49 (out of 90), respectively, as shown in Tables 5 and 6. In the preservation of human territory and the preservation of personal space, the highest average was related to " moving chairs or other items from the patient’s room without permission (3.44 ± 0.39 (out of 5)) " and " Respecting the privacy of patients during discharge (2.61 ± 0.31 (out of 5)) “, respectively. Furthermore, the overall average score for patients’ privacy preservation was 79.74 ± 14.53 (out of 150), indicating a moderate level of privacy preservation among the patients.

**Table 5:** Mean and standard deviation of the patient’s privacy compliance regarding preservation of human territory

Dimensions	Score range	Mean	Standard deviation
1. Guiding the patient in the ward and informing him about different parts	1–5	2.93	0.24
2. Introducing yourself to patients before performing care procedures		3.15	0.32
3. Explaining to patients before performing care procedures		2.97	0.19
4. Appropriate and correct answers to patients’ questions		3.23	0.27
5. Explanation about patients’ surgery		3.31	0.30
6. Entering the patient’s room without knocking		3.01	0.26
7. Creating a private environment during examination, injection and…		3.36	0.22
8. Carrying out care by a same-sex nurse in the operating room		3.12	0.31
9. Keeping the lights on at night and preventing the normal sleep of patients		3.34	0.44
10. The presence of noise in the ward and preventing the normal sleep of patients		3.33	0.19
11. Moving chairs or other items from the patient’s room without permission		3.44	0.39
12. Moving the bed when the patient is lying on the bed.		3.07	0.51
**Total**	**12–60**	**38.26**	**4.25**

**Table 6:** Mean and standard deviation of the patient’s privacy compliance regarding preservation of personal space

Dimensions	Score range	Mean	Standard deviation
1. Asking very private questions	1–5	2.21	0.29
2. Disrespect for patients’ equipment		2.29	0.28
3. Staff sitting on patients’ beds		2.11	0.24
4. Sudden awakening of patients		2.14	0.32
5. Ignoring the peace and quiet of patients		2.33	0.37
6. Hasty and careless treatment		2.31	0.24
7. Treatment in a harsh and impolite manner		2.43	0.18
8. Getting too close to the patient		2.46	0.25
9. Maintain patient coverage as much as possible		2.47	0.36
10. Looking directly into the eyes of patients		2.28	0.21
11. Calling patients by bed number		2.01	0.38
12. Disclosing private and confidential information		2.03	0.47
13. Disclosure of patient information in front of others		2.08	0.50
14. Attention to religious and belief principles		2.26	0.34
15. Psychological support for patients when they are afraid		2.51	0.29
16. Keeping patients covered in the operating room		2.45	0.17
17. Respecting the privacy of patients during discharge		2.61	0.31
18. Access to the phone or cell phone when necessary		2.50	0.42
**Total**	**18–90**	**41.48**	**6.49**

In accordance with Table 7, a statistically significant correlation was observed between nurses’ moral intelligence and cognitive flexibility with the level of patient privacy preservation. (Table 7).

**Table 7:** Correlation between ethical intelligence and cognitive flexibility of nurses with patient privacy protection

Variables		Dimensions	*P*-value^*^	*r*_p_^**^
**Patient privacy protection**	**Ethical intelligence**	Operating based on principles, values, and beliefs	< 0.001	0.313
		Honesty	< 0.001	0.207
		Resilience and advocacy for rights	< 0.001	0.379
		Commitment to promises	< 0.001	0.217
		Personal decision-making responsibility	< 0.001	0.284
		Admitting mistakes and failures	< 0.001	0.253
		Accepting responsibility for serving others	< 0.001	0.306
		Being actively interested in others	< 0.001	0.173
		The ability to forgive one’s own mistakes	< 0.001	0.272
		The ability to forgive mistakes of others	< 0.001	0.267
		**Total**	**< 0.001**	**0.414**
	**Cognitive flexibility**	Control	< 0.001	0.562
		Perception of multiple solutions	< 0.001	0.489
		Perception of justifications	< 0.001	0.249
		**Total**	**< 0.001**	**0.633**

* P-Value; Correlation is significant at the 0.05 level

** r_p_; Pearson correlation coefficient

The results of multiple linear regression analysis, aimed at determining the simultaneous impact of various dimensions of nurses’ moral intelligence on the level of patient privacy preservation, indicated that the significant variables in the model determined using the Enter method were, in order of importance: “perseverance and insistence on rights, action based on principles, values, and beliefs, responsibility for personal decisions, taking responsibility for serving others, the ability to forgive one’s own mistakes, the ability to forgive the mistakes of others, honesty, acknowledgment of mistakes and failures, commitment to promises, and empathy for others.” This suggests that these dimensions had a meaningful impact on the level of patient privacy preservation.

Additionally, regression analysis to determine the simultaneous effect of different dimensions of nurses’ cognitive flexibility on the level of patient privacy preservation indicated a significant influence of the dimensions of these variables, namely perception of control, perception of multiple solutions, and perception of justifications.

The β values for the influential variables, which indicate the priority of their impact on the level of patient privacy preservation, are presented in Table 8. Furthermore, this analysis showed that the adjusted model determination coefficient (R^2^ Adjusted) for the level of patient privacy preservation based on the variables of nurses’ moral intelligence and cognitive flexibility is 0.51 and 0.62, respectively. This means that 51% and 62% of the variation in the patient privacy preservation score can be explained by the variables in the model.

**Table 8:** Factors affecting patients’ privacy compliance using multiple linear regression model

Variable			Unstandardized coefficients		Standardized coefficient β	P-value^*^
			B	Std. Error		
The patients’ privacy compliance	**---**	**(Constant)**	**1.086**	**0.298**	**---**	**<0.001**
	X_1_	Resilience and advocacy for rights	0.540	0.087	0.463	**<0.001**
	X_2_	Operating based on principles, values, and beliefs	0.454	0.078	0.420	**<0.001**
	X_3_	Personal decision-making responsibility	0.410	0.065	0.389	**<0.001**
	X_4_	Accepting responsibility for serving others	0.393	0.078	0.351	**<0.001**
	X_5_	The ability to forgive one’s own mistakes	0.301	0.055	0.297	**0.001**
	X_6_	The ability to forgive mistakes of others	0.287	0.097	0.243	**0.002**
	X_7_	Honesty	0.275	0.063	0.211	**0.004**
	X_8_	Admitting mistakes and failures	0.263	0.094	0.207	**0.005**
	X_9_	Commitment to promises	0.242	0.066	0.198	**0.005**
	X_10_	Being actively interested in others	0.237	0.101	0.184	**0.01**
	**---**	**(Constant)**	**1.317**	**0.376**	**---**	**0.001**
	X_11_	Control	0.580	0.042	0.530	**<0.001**
	X_12_	Perception of multiple solutions	0.511	0.045	0.435	**<0.001**
	X_13_	Perception of justifications	0.373	0.028	0.242	**<0.001**

* P-Value; Correlation is significant at the 0.05 level

The linear equations for the patient privacy preservation score based on the variables in the explanatory model are as follows:

$$\eqalign{{\rm{Y}}\,{\rm{ = }}\, & {\rm{1}}{\rm{.086}}\,{\rm{ + }}\,{\rm{0}}{\rm{.540}}{{\rm{X}}_1}\, + \,0.454{{\rm{X}}_2}\, + \,0.410{{\rm{X}}_3} \cr & \, + \,0.393{{\rm{X}}_4}\, + \,0.301{{\rm{X}}_5}\, + \,0.287{{\rm{X}}_6}\, + \,0.275{{\rm{X}}_7} \cr & \, + \,0.263{{\rm{X}}_8}\, + \,0.242{{\rm{X}}_9}\, + \,0.237{{\rm{X}}_{10}} \cr}$$

(Where Y represents the patient privacy preservation score, and X_1_ to X_10_ are the influential variables, with moral intelligence of nurses as the reference variable).

$${\rm{Y}}\,{\rm{ = }}\,{\rm{1}}{\rm{.317}}\,{\rm{ + }}\,{\rm{0}}{\rm{.580}}{{\rm{X}}_{11}}\, + \,0.511{{\rm{X}}_{12}}\, + \,0.373{{\rm{X}}_{13}}$$

(Where Y represents the patient privacy preservation score, and X_11_ to X_13_ are the influential variables, with cognitive flexibility of nurses as the reference variable).

## Discussion

The study estimated nurses’ ethical intelligence at a moderate level. Contrasting findings were observed in other studies: Sadri Damirchi et al. (2019) reported weak ethical intelligence [52], Amini et al. (2015) found it above average [53], and Saied et al. (2018) and Nehrir et al. (2015) reported it at a moderate level [54, 55]. Further, Gorzin et al. (2015), Khajavi et al. (2020), and Tarazoj et al. (2017) indicated good to high levels [56–58], while Dehghani et al. (2022) found very good scores in specialized care [59]. Variations in findings may be attributed to differences in research settings and populations. Given the moderate ethical intelligence in this study, enhancing nurses’ capabilities through training is essential, as it benefits stakeholders’ interests, workplace understanding, team performance, commitment, efficiency, and effectiveness [55].

The study revealed that ethical intelligence increases with age, corroborated by Nehrir et al. (2015), Dehghani et al. (2022), Ansari Shahidi et al. (2018), Majidi et al. (2018), Mohammadi et al. (2014), and Khajavi et al. (2020) [8, 55, 57, 59–61]. Lapointe and Langlois (2007) and Bahrami et al. (2012) also reported a positive relationship between age and ethical intelligence [47, 62]. However, Saied et al. (2018) and Amini et al. (2015) found no significant correlation [53, 54]. This suggests that ethical intelligence, being acquirable, likely increases with age and experience, helping nurses distinguish between correct and incorrect actions through repeated exposure to various challenges.

Female nurses in this study exhibited higher ethical intelligence than males, supported by Nehrir et al. (2015) and Majidi et al. (2018) [55, 61], but contradicted by Amini et al. (2015), Ansari Shahidi et al. (2018), and Khajavi et al. (2020) who found no gender difference [53, 57, 60]. Saied et al. (2018) reported higher ethical intelligence in men [54]. These differences may stem from personality traits and women’s dual professional and household roles. The study also found that ethical intelligence decreased with an increase in shifts and patient load, consistent with Saied et al. (2018) who observed a negative relationship between moral intelligence and overtime hours [54]. This might result from the emotional and physical demands of clinical environments, affecting ethical decision-making. Married nurses demonstrated higher ethical intelligence, potentially due to increased responsibilities and sensitivity to ethical issues. However, Saied et al. (2018) and Khajavi et al. (2020) reported no significant difference based on marital status [54, 57]. Nurses with a master’s degree had higher ethical intelligence, as also found by Saied et al. (2018), Majidi et al. (2018), Pierce and Sweeney (2010), Hernandez and McGee (2013), and Goldman and Tabak (2010) [54, 61, 63–65]. Conversely, Amini et al. (2015), Nehrir et al. (2015), and Khajavi et al. (2020) found no significant relationship with education level [53, 55, 57]. The variations could be due to different study environments, suggesting that ethical intelligence evolves with education and age.

Cognitive flexibility among nurses was also moderate, aligning with Mirkamali et al. (2021), Sadri Damirchi et al. (2019), Shams (2020), and Veismoradi et al. (2023) [52, 66–68]. Bahari (2021) and Fallah Madvari et al. (2022) found high levels [50, 69], while Feshangchi and Ranjbar Noushari (2020) reported low levels [70]. Differences may be due to varying hospital settings and personality traits. Cognitive flexibility helps nurses manage stress and maintain positive emotions, aiding in patient care. Cognitive flexibility increased with age in this study, contradicting Entezari et al. (2018) who found it decreases with age [71], but aligning with Holmberg et al. (2020) who found no significant relationship [72]. Older nurses may adapt better to changing environments, enhancing their cognitive flexibility and control over patient care.

Patient privacy preservation by nurses was moderate, similar to findings by Dehghani et al. (2016), Hajbaghery et al. (2014), and Sabet Sarvestani et al. (2019) [73–75]. Moogahi et al. (2020) reported high levels [76], while Rasti et al. (2014) found it moderate [51]. Differences may stem from varying patient expectations and cultural norms. Privacy preservation increased with patient age, differing from Rasti et al. (2014) and Sabet Sarvestani et al. (2019) who found no significant difference [51, 75]. Zihaghi et al. (2016) reported an inverse relationship [77], while Harorani et al. (2017) found a significant correlation [78]. Variations may be due to different cultural backgrounds and societal norms. Female patients perceived higher privacy preservation than males, supported by Noorian et al. (2016) [79], but contradicted by Aghajani and Dehghannayeri (2010) and Rasti and Jahanpour (2014) [51, 80]. Differences may arise from varying perceptions and expectations between genders. Urban residents reported higher privacy preservation than rural ones, aligning with Rasti and Jahanpour (2014) [51] but contradicting Zirak et al. (2015) [81]. Differences in awareness and expectations may contribute to these variations. Patients with higher education perceived better privacy preservation, supported by Rasti and Jahanpour (2014) and Harorani et al. (2017) [51, 78], but not by Noorian et al. (2016) or Zirak et al. (2015) [79, 81]. Education may influence patients’ expectations and perceptions of privacy.

A positive correlation was found between nurses’ emotional intelligence, cognitive flexibility, and patient privacy preservation, aligning with Kalantari et al. (2020) and Mahdiyoun et al. (2017) [82, 83]. Higher emotional and cognitive skills in nurses enhance their sensitivity to ethical and privacy issues. Regression analysis identified emotional intelligence dimensions (resilience, principle-based action, responsibility, honesty, commitment) and cognitive flexibility dimensions (control perception, perception of multiple solutions, perception of justifications) as significant predictors of patient privacy protection. Sadeghi et al. (2015) and Mohammadi et al. (2014) also found similar predictors for patient satisfaction and education [8, 16]. Nazem et al. (2022) noted a direct relationship between cognitive flexibility and ethical sensitivity [84]. Enhanced cognitive flexibility aids nurses in managing ethical decisions, fostering trust and better patient care. In conclusion, ethical and cognitive skills in nurses are crucial for patient care quality. Enhancing these skills through targeted training and education can improve patient satisfaction and ethical standards in healthcare settings. Future studies should explore these relationships further across diverse healthcare environments.

## Conclusion

The level of patient privacy protection is influenced by the level of ethical intelligence and cognitive flexibility of nurses. As the dimensions of ethical intelligence and cognitive flexibility of nurses increase, the level of patient privacy protection also increases. Therefore, to strengthen and improve patient privacy protection, it is necessary to take action to enhance the ethical intelligence and cognitive flexibility of nurses through educational, welfare, managerial, motivational, and occupational policies.

### Limitations

The limitations of this study include the cross-sectional examination, self-reporting data collection, which may influence the reports of nurses and patients, and the generalizability of the findings to other communities and cultures. Therefore, it is recommended to conduct interventional, qualitative, and longitudinal studies in research environments with different cultures and other hospitals.

### Recommendations and practical application

The findings of this study, besides providing the status of nurses’ ethical intelligence and cognitive flexibility and the level of patient privacy protection in hospitals, help to better understand the impact of nurses’ ethical intelligence and cognitive flexibility on patient privacy protection. Therefore, hospital managers and policymakers can use the findings of this study as evidence and documentation to influence and improve the status of patient privacy protection by considering nurses’ ethical intelligence and cognitive flexibility.

## Data Availability

All the data is presented as a part of tables or figures. Additional data can be requested from the corresponding author.

## References

[1] Poorchangizi B, Farokhzadian J, Abbaszadeh A, Mirzaee M, Borhani F. The importance of professional values from clinical nurses’ perspective in hospitals of a medical university in Iran. BMC Med Ethics. 2017;18(1):1–7. 28249603 10.1186/s12910-017-0178-9 PMC5333397

[2] Mehralian G, Yusefi AR, Ahmadinejad P, Bahmaei J, Bordbar S. Investigating the professional commitment and its correlation with patient safety culture and patient identification errors: Evidence from a cross-sectional study from nurses’ perspectives. Open Public Health J. 2024;17(1).

[3] Song J. Ethics education in nursing: challenges for nurse educators. Kaitiaki Nurs Res. 2018;9(1):12–7.

[4] Brännmark J. The independence of medical ethics. Med Health Care Philos. 2019;22(1):5–15. 29770916 10.1007/s11019-018-9842-1 PMC6394499

[5] Waldemar AK, Esbensen BA, Korsbek L, Petersen L, Arnfred S. Recovery orientation in mental health inpatient settings: inpatient experiences? Int J Ment Health Nurs. 2018;27(3):1177–87. 29359397 10.1111/inm.12434

[6] Khammarnia M, Setoodezadeh F, Ansari-Moghadam A, Yusefi A, Eskandari M, Ranjbar AA, Peyvand M. Relationship between health literacy of cancer patients and shared clinical decision-making in a Middle East country. Epidemiol Biostatistics Public Health. 2018;15(1):1–11.

[7] Yusefi AR, Sarvestani SR, Kavosi Z, Bahmaei J, Mehrizi MM, Mehralian G. Patients’ perceptions of the quality of nursing services. BMC Nurs. 2022;21(1):1–10. 35624460 10.1186/s12912-022-00906-1 PMC9137069

[8] Mohammadi S, Nakhaei N, Borhani F, Roshanzadeh M. Moral intelligence in nursing: a cross-sectional study in East of Iran. Iran J Microbiol. 2014;6(5):58–66.

[9] Mohamadi J, Ghazanfari F, Azizi A. Relationship between moral intelligence and nurses’ quality of Work Life. IJN. 2014;27(90–91):54–64.

[10] Mehralian G, Yusefi AR, Dastyar N, Bordbar S. Communication competence, self-efficacy, and spiritual intelligence: evidence from nurses. BMC Nurs. 2023;22(1):1–9. 37024881 10.1186/s12912-023-01262-4 PMC10077309

[11] Manzari Tavakoli H, Salajeghe S, Sheikhi A. Development and psychometric evaluation of nurses’ Moral Intelligence Scale in Kerman University of Medical Sciences Hospitals. J Health Promotion Manage. 2019;8(5):9–17.

[12] Yusefi AR, Ebrahim Z, Zargar Balaye Jame S, Bastani P. Workload and its associated factors among nurses in teaching hospitals of Shiraz University of Medical Sciences in 2017. J Res Dev Nurs Midwifery. 2019;16(1):81–91. 10.4103/ijnmr.IJNMR_205_17 PMC6298171 30622582

[13] Khosravani M, Khosravani M, Borhani F, Mohsenpour M. The relationship between moral intelligence and organizational commitment of nurses. Clin Ethics. 2020;15(3):126–31. 10.1177/1477750920908008

[14] Habibzade H, Ahmadi F, Vanaki Z. Ethics in professional nursing in Iran. Iran J Med Ethics Hist Med. 2010;3(5):26–36.

[15] Lau HC, Idris MA. Soft foundations of the critical success factors onetime implementation in Malaysia. TQM Magazine. 2005;13(4):515–52.

[16] Sadeghi A, Adeli Z, Shamsaei F, Moghim Beigi A. Relationship between nurses’ moral intelligence and patient’ satisfaction from nursing care. Q J Nurs Manage. 2015;4(3):65–76.

[17] Yousefi P, Heshmati H. Moral Intelligence and its position in nursing Profession. J Dev Strategies Med Educ. 2016;2(2):65–73.

[18] Haahr A, Norlyk A, Martinsen B, Dreyer P. Nurses experiences of ethical dilemmas: a review. Nurs Ethics. 2020;27(1):258–72. 30975034 10.1177/0969733019832941

[19] Machado DA, Figueiredo NM, Velasques LD, Bento CA, Machado WC, Vianna LA. Cognitive changes in nurses working in intensive care units. Revista brasileira de enfermagem. 2018;71:73–9. 29324947 10.1590/0034-7167-2016-0513

[20] Izquierdo A, Brigman JL, Radke AK, Rudebeck PH, Holmes A. The neural basis of reversal learning: an updated perspective. Neuroscience. 2017;345:12–26. 26979052 10.1016/j.neuroscience.2016.03.021 PMC5018909

[21] Kılıç Z, Uzdil N, Günaydın Y. The effect of cognitive flexibility in nurses on attitudes to professional autonomy. Nurs Ethics. 2023 Aug;21:09697330231174533. 10.1177/09697330231174533 37602374

[22] Braem S, Egner T. Getting a grip on cognitive flexibility. Curr Dir Psychol Sci. 2018;27(6):470–6. 30555214 10.1177/0963721418787475 PMC6291219

[23] Medaglia JD, Huang W, Karuza EA, Kelkar A, Thompson-Schill SL, Ribeiro A, Bassett DS. Functional alignment with anatomical networks is associated with cognitive flexibility. Nat Hum Behav. 2018;2(2):156–64. 30498789 10.1038/s41562-017-0260-9 PMC6258039

[24] Laureiro-Martínez D, Brusoni S. Cognitive flexibility and adaptive decision‐making: evidence from a laboratory study of expert decision makers. Strateg Manag J. 2018;39(4):1031–58. 10.1002/smj.2774

[25] Mohammadi M, Vaisi-Raygani A, Jalali R, Salari N. Prevalence of job stress in nurses working in Iranian hospitals: a systematic review, meta-analysis and meta- regression study. J Health Saf Work. 2020;10(2):119–28.

[26] Leka S, Hassard J, Yanagida A. Investigating the impact of psychosocial risks and occupational stress on psychiatric hospital nurses’ mental well-being in Japan. J Psychiatr Ment Health Nurs. 2012;19(2):123–31. 22070548 10.1111/j.1365-2850.2011.01764.x

[27] Haspel RL, Lin Y, Fisher P, Ali A, Parks E. Development of a validated exam to assess physician transfusion medicine knowledge. Transfusion. 2014;54(5):1225–30. 24117860 10.1111/trf.12425

[28] Gustavsson JP, Hallsten L, Rudman A. Early career burnout among nurses: modelling a hypothesized process using an item response approach. Int J Nurs Stud. 2010;47(7):864–75. 20070968 10.1016/j.ijnurstu.2009.12.007

[29] Christensen SS, Wilson BL, Edelman LS. Can I relate? A review and guide for nurse managers in leading generations. J Nurs Adm Manag. 2018;26(6):689–95. 10.1111/jonm.12601 29380917

[30] Yang NY, Baek JY, Kim KE, Park JE. Influence of cognitive flexibility, resilience, and Professional Quality of Life on Job Embeddedness of nurses. J Korean Acad Soc Home Health Care Nurs. 2023;30(2):174–82.

[31] Kent W, Hochard KD, Hulbert-Williams NJ. Perceived stress and professional quality of life in nursing staff: how important is psychological flexibility? J Context Behav Sci. 2019;14:11–9. 10.1016/j.jcbs.2019.08.004

[32] Polat Ş, Afşar Doğrusöz L, Yeşil A. The relationship between cognitive flexibility and happiness among nurses. Perspect Psychiatr Care. 2022;58(4):2862–71. 35904443 10.1111/ppc.13134

[33] Khorshidi G, DashtBozorgi Z. Relationship of dark triad of personality, sexual assertiveness and cognitive flexibility with marital burnout in female nurses. Iran J Nurs Res. 2019;14(1):65–71.

[34] Mahdavi Neysiani Z, Asgari M, Hosseni J, Tamananlo Z. [The relationship between cognitive flexibility and burnout and occupational injuries in female emergency nurses (Persian). Paper presented at: 7th International Conference on Psychology nand Social Sciences. 16 February 2017; Tehran, Iran. https://civilica.com/doc/639010/certificate/print/.

[35] Culha Y, Acaroglu R. The relationship amongst student nurses’ values, emotional intelligence and individualised care perceptions. Nurs Ethics. 2019;26(7–8):2373–83. 30336766 10.1177/0969733018796682

[36] Khosravani M, Borhani F, Loghmani L, Mohsenpour M. Ethical sensitivity relationship with communication skills in Iranian nursing managers. Int J Pharm Res. 2018;10(3):143–7.

[37] Stokes F, Palmer A. Artificial intelligence and robotics in nursing: ethics of caring as a guide to dividing tasks between AI and humans. Nurs Philos. 2020;21(4):e12306. 32609420 10.1111/nup.12306

[38] Luo E, Bhuiyan MZ, Wang G, Rahman MA, Wu J, Atiquzzaman M, Privacyprotector. Privacy-protected patient data collection in IoT-based healthcare systems. IEEE Commun Mag. 2018;56(2):163–8. 10.1109/MCOM.2018.1700364

[39] Aboujaoude E. Protecting privacy to protect mental health: the new ethical imperative. J Med Ethics. 2019;45(9):604–7. 31123190 10.1136/medethics-2018-105313

[40] Abbasinia M, Ahmadi F, Kazemnejad A. Patient advocacy in nursing: a concept analysis. Nurs Ethics. 2020;27(1):141–51. 31109243 10.1177/0969733019832950

[41] Bijani M, Tehranineshat B, Torabizadeh C. Nurses’, nursing students’, and nursing instructors’ perceptions of professional values: a comparative study. Nurs Ethics. 2019;26(3):870–83. 28905676 10.1177/0969733017727153

[42] McCarthy S, O’Raghallaigh P, Woodworth S, Lim YL, Kenny LC, Adam F. An integrated patient journey mapping tool for embedding quality in healthcare service reform. J Decis Syst. 2016;25(sup1):354–68. 10.1080/12460125.2016.1187394

[43] Sadeghi T, Dehghan Nayyeri N, Karimi R. Nursing-patient relationship: a comparison between nurses and adolescents perceptions. IJMEHM. 2011;4(3):69–78.

[44] Kuzu N, Ergin A, Zencir M. Patients’ awareness of their rights in a developing country. Public Health. 2006;120(4):290–6. 16476454 10.1016/j.puhe.2005.10.014

[45] Erdil F, Korkmaz F. Ethical problems observed by student nurses. Nurs Ethics. 2009;16(5):589–98. 19671645 10.1177/0969733009106651

[46] Nayeri N, Aghajani M. Patients’ privacy and satisfaction in the emergency department: a descriptive analytical study. Nurs Ethics. 2010;17(2):167–77. 20185441 10.1177/0969733009355377

[47] Bahrami MA, Asami M, Fatehpanah A, Dehghani Tafti A, Ahmadi Tehrani G. Moral intelligence status of the faculty members and staff of the Shahid Sadoughi University of Medical Sciences of Yazd. Iran J Med Ethics Hist Med. 2012;5(6):81–95.

[48] Lennick D, Kiel F. Moral intelligence: enhancing business performance and leadership success in turbulent times. Pearson Prentice Hall; 2011.

[49] Dennis JP, Vander Wal JS. The cognitive flexibility inventory: instrument development and estimates of reliability and validity. Cogn Ther Res. 2010;34(3):241–53. 10.1007/s10608-009-9276-4

[50] Bahari Z. The mediating role of cognitive flexibility in the relationship between job stress and psychological wellbeing of nurses. Iran J Nurs. 2021;34(133):16–27.

[51] Jahanpour F, Rasti R. Viewpoints of nurses and patients on paying respect to the privacy of patients in Care. J Mazandaran Univ Med Sci. 2014;24(111):34–42.

[52] Sadri Damirchi S, Mohammadi N, Rahimi Zarj Abad N. Prediction of mental health based on moral intelligence and cognitive flexibility in nursing students. J Med Ethics. 2018;12(43):1–10.

[53] Amini M, Rahimi H, Godali H, Montazer M. Study the Status of Moral Intelligence in nurses across Kashan Hospitals in 2015. J Educ Ethics Nurs. 4(1):60–6.

[54] Saied Y, Afaghi E, Tabanejad Z, Najafloo M. The relationship between Moral Intelligence and Demographic Characteristics of Nurses in the Intensive Care Units. Military Caring Sci. 2018;4(4):281–7. 10.29252/mcs.4.4.281

[55] Nehrir B, Saeid Y, Ebadi A, Najafloo M, Khoshab H, Mahmoodi H, et al. A comparison of the moral intelligence of nurses in civilian and military hospitals. Iran J Med Ethics History Med. 2015;7(6):59–68.

[56] Asgari Tarazoj A, Mohammadzadeh A, Hejazi S. Relationship between moral intelligence and anger among nurses in emergency units of hospitals affiliated to Kashan University of Medical Sciences. J Health Care. 2018;19(4):262–71.

[57] Khajavi Z, Vaezzadeh N, Mousavinasab SN, Azimi Lolaty H. Relationship between ethical intelligence and professional behavior in nurses. J Mazandaran Univ Med Sci. 2020;30(185):86–95.

[58] Gorzin H, Nehrir B, Moradian ST, Spandi M. Nurses’ Moral Intelligence in a selected Military Hospital. A descriptive-Analytical Study. J Crit Care Nurs. 2019;12(2):28–34.

[59] Dehghani M, Mousazadeh N, Hakimi H. Moral intelligence and demographic characteristics of the nurses working in intensive care units: a descriptive-correlational study. Nurs Midwifery J. 2022;20(3):170–7. 10.52547/unmf.20.3.170

[60] Ansari Shahidi M, Tat M, Maleki S. The role of ethical intelligence and professional value in predicting nurses’ resilience. Yafteh. 2018;20(3):48–58.

[61] Majidi SA, Kouchakzadeh S, Safarmohammadi H, Leyli EK. Assessment of hospital nurses’ moral intelligence: a cross-sectional study in Guilan province, North of Iran. Shiraz E-Medical J. 2018;19(10):e62299. 10.5812/semj.62299

[62] Langlois L, Lapointe C. Ethical leadership in Canadian school organizations: tensions and possibilities. Educational Manage Adm Leadersh. 2007;35(2):247–60. 10.1177/1741143207075391

[63] Pierce B, Sweeney B. The relationship between demographic variables and ethical decision making of trainee accountants. Int J Auditing. 2010;14(1):79–99. 10.1111/j.1099-1123.2009.00404.x

[64] Hernandez T, McGee RW. Ethical attitudes toward taking a bribe: a study of four European countries. Available SSRN 2199245. 2013.

[65] Goldman A, Tabak N. Perception of ethical climate and its relationship to nurses’ demographic characteristics and job satisfaction. Nurs Ethics. 2010;17(2):233–46. 20185447 10.1177/0969733009352048

[66] Mirkamali FS, Khosravi SA, Shayegan M, Imani F, Afshar EK. The effectiveness of Positivist psychology training on cognitive flexibility and psychological hardiness of nurses. J Nurs Educ (JNE). 2021;10(3):1–10.

[67] Shams S. Predicting Covid Disease-19 anxiety based on perceived stress and anxiety sensitivity in nurses: the mediating role of cognitive flexibility. J Health Promotion Manage. 2022;11(3):1–14.

[68] Veismoradi M, Borjali A, Rafezi Z. Predicting nurses’ quality of life based on coronavirus-induced anxiety and cognitive flexibility. Clin Psychol Personality. 2023;21(1):171–80.

[69] Fallah Madvari R, Zare Sakhvidi MJ, Sefidkar R, Jafari Nodoushan M. Investigating corona disease anxiety in nurses and its relationship with cognitive flexibility: a case study. Archives Occup Health. 2022;6(2):1243–9.

[70] Feshangchi P, Ranjbar Noushari F. The correlation of psychological flexibility, type D personality and perceived Social Support with Job Tension in nurses. Iran J Psychiatric Nurs. 2020;8(1):39–48.

[71] Entezari M, Shamsipour Dehkordi P, Sahaf R. Effect of physical activity on cognitive flexibility and perfectionism in the Elderly. Salmand: Iran J Ageing. 2018;12(4):402–13. 10.21859/sija.12.4.402

[72] Holmberg J, Kemani MK, Holmström L, Öst L-G, Wicksell RK. Psychological flexibility and its relationship to distress and work engagement among intensive care medical staff. Front Psychol. 2020;11:603986. 33250832 10.3389/fpsyg.2020.603986 PMC7672021

[73] Dehghani F, Abbasinia M, Heidari A, Mohammad Salehi N, Firoozi F, Shakeri M. Patient’s view about the protection of privacy by Healthcare Practitioners in Shahid Beheshti Hospital, Qom, Iran. Iran J Nurs. 2016;28(98):58–66. 10.29252/ijn.28.98.58

[74] Hajbaghery MA, Zehtabchi S. Evaluation of elderly patients’ privacy and their satisfaction level of privacy in selected hospitals in esfahan. Med Ethics J. 2014;8:97–120.

[75] Sabet Sarvestani R, Khani Jeyhooni A. Comparison of the viewpoints of the operating room staff and those of patients on the degree of respecting patients’ privacy. Educ Ethic Nurs. 2019;8(1):14–20. 10.52547/ethicnurs.8.1.2.14

[76] Moogahi S, Tajik M, Cheraghi M, Jamshidi F. Evaluation of privacy level among Elderly patients in the Educational and Medical Centers of Ahvaz Jundishapur University of Medical Sciences in 2019. Iran J Med Ethics History Med. 2020;13(0):607–18.

[77] Zihaghi M, Saber S, Nouhi E. Respect for privacy by nurses from the perspective of the elderly hospitalized in internal and surgical wards. Medical-Surgical Nurs J. 2016;5(3).

[78] Harorani M, Pakniat A, Jadidi A, Sadeghi H, Varvanifarahani P, Golitaleb M. The extent of maintaining the privacy of patients hospitalized in Emergency Departments of hospitals affiliated with Arak University of Medical Sciences; a cross-sectional study. Iran J Emerg Med. 2017;4(4):158–63.

[79] Noorian K, Hashemi H, Salehi Z, Rahimi Madiseh M. Comparison of operation room staffs and patients perspectives from patient privacy in the operating room. J Clin Nurs Midwifery. 2016;5(1):47–57.

[80] Dehghannayeri N, Aghajani M. Protecting patients’ privacy by Medical Team and its relation to patients’ satisfaction. J Hayat. 2010;16(1):13–22.

[81] Zirak M, Ghafourifard M, Aghajanloo A, Haririan H. Respect for patient privacy in the teaching hospitals of Zanjan. J Med Ethics History Med. 2015;8(1):79–89.

[82] Kalantari Z, Varjoshani NJ, Hanifi N, Fallah R. The relationship between moral intelligence of emergency personnel with the level of respect for patients’ privacy in the emergency departments, Zanjan, Iran, 2018. J Military Med. 2020;22(7):760–70.

[83] Mahdiyoun SA, Pooshgan Z, Imanipour M, Razaghi Z. Correlation between the nurses, moral sensitivity and the observance of patients’ rights in ICUs. Med Ethics J. 2017;11(40):7–14. 10.21859/mej-11407

[84] Nazem M, Rezapour Mirsalehi Y, Arianpour HR. The Mediating Role of Cognitive Flexibility in the relationship between Professionl values and Moral sensitivity among nurses during the Corona Epidemic. J Med Ethics. 2022;16(47):e1.

